# Brandt's vole (*Lasiopodomys brandtii*) affects the dominant position of three gramineous species by altering defense traits and interspecific competition

**DOI:** 10.1002/ece3.70086

**Published:** 2024-08-01

**Authors:** Yanjin Xie, Yongle Hua, Jiading Zhang, Wanhong Wei, Baofa Yin

**Affiliations:** ^1^ College of Bioscience and Biotechnology Yangzhou University Yangzhou China

**Keywords:** dominant position, escape characteristics, functional traits, plant defense, resistance, tolerance

## Abstract

Rodents can cause considerable changes in plant community composition. However, relationships between shifts in species dominance and plant functional traits caused by rodents have seldom been investigated, especially for belowground functional traits. In this study, a set of enclosures was constructed to analyze the effects of 10 years of Brandt's voles' activities on the defense strategies and dominant position changes of three gramineous plants (*Leymus chinensis*, *Stipa krylovii*, and *Cleistogenes squarrosa*) in Inner Mongolia. Here, we measured the dominance, biomass, and fourteen functional traits of three plants. The effects of Brandt's voles on dominance, biomass, and functional traits were analyzed, and then we explored the effect of functional traits on plant dominance by using the structural equation model. Results showed that long‐term feeding by Brandt's voles resulted in a significant decrease in the dominance of *L. chinensis* and *S. krylovii*, whereas *C. squarrosa* was positively affected. The belowground biomass of *L. chinensis* and *S. krylovii* was higher in the vole treatment, which showed that they were increasing their escape characteristics. The leaf thickness of *L. chinensis* and the leaf C:N ratio of *S. krylovii* significantly increased, while the specific leaf area of *C. squarrosa* significantly decreased. All three gramineous showed increased resistance traits in response to Brandt's voles, which positively affected their dominance. Tolerance‐related traits of *S. krylovii* significantly increased, with the increasing growth rate of root length contributing to enhancing its dominance. We highlight that selective feeding by rodents led to the selection of different defense strategies by three gramineous plants, and that changes in biomass allocation and functional traits in the different species affected plant dominance, driving changes in the plant communities.

## Graphical abstract

Herbivores changed plant community composition, with unpreferred plant species becoming the first dominant species. Feeding pressure and competition pressure drove the change in plant functional traits; different plants showed inconsistent defense strategies, which led to the change in plant dominance.

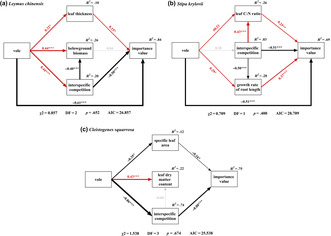

## 1 INTRODUCTION

The interactions between animals and plants have always been a key focus of ecological research (Loayza et al., 2020; Parra et al., 2022; Sharp Bowman et al., 2017). Selective feeding by herbivores can limit or promote the growth and development of different plants, change interspecific relationships among plants, and change the composition and structure of plant communities (Ji et al., 2021; Jia et al., 2018). Plants have evolved various defensive strategies to deal with disturbance from herbivores (de Bobadilla et al., 2022; Wari et al., 2022). Plants improve their tolerance to herbivores by increasing photosynthetic rates, compensating for growth, or mobilizing aboveground resource allocation (Barton, 2013; Capó et al., 2021; Koch et al., 2016). Plants have also evolved physical and chemical defenses that increase their resistance to feeding activities (Salgado‐Luarte et al., 2022; Wari et al., 2022). Plants can also escape herbivores by altering growth patterns or energy allocation, such as increasing belowground biomass during overgrazing (Dai et al., 2019; Rhodes et al., 2018).

Selective feeding by herbivores changes the resource allocation mode of plants. This results in changes in the morphological, physiological, and other functional traits of the aboveground organs of plants in response to herbivores (He et al., 2021). For example, plant height and leaf dry matter content positively correlate with plant light resource acquisition ability and competitiveness, improving plants' tolerance (Smart et al., 2017; Zhang et al., 2018; Zhang, Zhu, et al., 2019). Leaf thickness is positively correlated with blade toughness, and specific leaf area and leaf C:N ratio affect plant palatability; these traits play a role in deterring herbivores (Agrawal & Fishbein, 2006; Pérez‐Harguindeguy et al., 2013; Zvereva et al., 2020). Except, herbivores also affect belowground functional traits, such as morphology, spatial distribution, and configuration of plant roots (Klumpp et al., 2009). Such as the increase in the growth rate of root length and root surface area density, and the decrease in average root diameter improves the absorption efficiency of soil water and nitrogen, which is conducive to the rapid growth of plants to tolerate the feeding pressure of herbivores (Chen, Xiong, & Cheng, 2021; Wang et al., 2021). Plant defense strategies against herbivores feeding in terms of their aboveground or belowground functional traits have been studied (Danet et al., 2017; Li et al., 2017; Smith et al., 2014), but compared with aboveground traits, belowground traits are understudied (Bergmann et al., 2017; He et al., 2021).

Besides that, plants must compete with other plants to obtain light, water, nutrients, and other resources as well as resist pressure from herbivore feeding (Lind et al., 2013). Interspecific competition can change the plant height, diameter, and surface area of root (Boege, 2010; Colom & Baucom, 2020). Therefore, plant defense strategies that are co‐regulated by herbivores and interspecific competition are worth exploring (Ballhorn et al., 2014). However, until now, most studies focused on the interaction process between grazing livestock or insects, and studies involved the relationship between small phytophagous rodents and plants were lacking, especially in the grassland system (Jiang et al., 2023; Liu et al., 2023; Zhang, van Doan, et al., 2019).

Brandt's vole (*Lasiopodomys brandtii*) is a typical small mammalian rodent, widely distributed in the grasslands of China, Mongolia, and Russia (Avirmed et al., 2016). It mainly feeds on grassland dominant grass plants and plays a significant role in promoting the nutrient cycling of grassland ecosystems (Hua et al., 2022). Its activities, such as digging and feeding behaviors, lead to the decline or disappearance of some plants and changes in the interspecific competition relationships and community structures of plants (Cui et al., 2020; Yin et al., 2017). If Brandt's vole overconsumes high‐quality forage, it may accelerate grassland degradation, threaten the balance of grassland ecosystems, and affect the development of local livestock (Li et al., 2023). Therefore, to maintain the stability of local ecosystems, it is of considerable importance to determine the effect of Brandt's voles on plants and the response strategies of plants to their activities. And we look forward to providing some insights into the understanding of interactions between small phytophagous rodents and plants in the grassland system.

In the present study, the response strategies, adaptation mechanisms, and changes in dominance of three common gramineous plants (*Leymus chinensis*, *Stipa krylovii*, and *Cleistogenes squarrosa*) on the activities of Brandt's voles were investigated using a large‐scale field fence control experiment. The main research questions were as follows: (1) After 10 years of feeding activity by Brandt's vole, did the dominance of the three gramineous plants change? (2) How did the three gramineous plants respond to disturbance by Brandt's vole through the growth–defense tradeoffs of aboveground and belowground functional traits? (3) How does Brandt's vole regulate the dominant positions of the three gramineous plants?

## 2 MATERIALS AND METHODS

### 2.1 Study site

This study was conducted at the Research Station of Animal Ecology, which is located in Maodeng pasture (44°11′ N, 116°27′ E; at 1100 m elevation), 38 km northeast of Xilinhot, Inner Mongolia, China. The mean annual temperature of the region is 0.9°C, and the mean annual precipitation at the study site is 241 mm, with 75% occurring during the growing season (May–August). The soil type is typical chestnut soil. The area is a typical steppe habitat with chestnut soil, dominated by the perennial grasses *L. chinensis*, *S. krylovii*, and *C. squarrosa*. Other plant species include *Paraphlomis lanceolata*, *Carex tristachya*, and *Chenopodium aristatum* (Cui et al., 2020).

### 2.2 Experimental design

**FIGURE 1:**
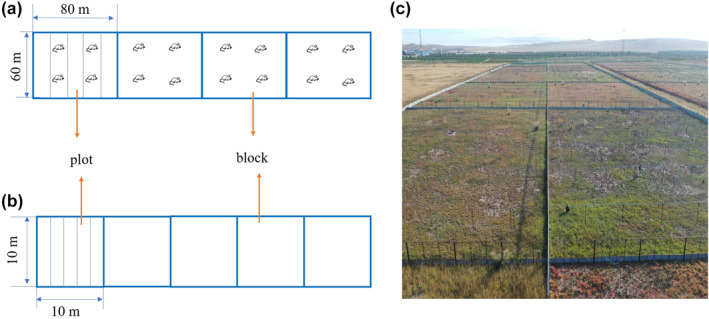
Experimental design diagrams (a) four enclosures (60 m × 80 m) with vole treatment, 13 or 15 vole breeding pairs were introduced into each enclosure (block) as founding populations, and each block was divided into five plots; (b) five enclosures (10 m × 10 m) with control treatment, each block was divided into five plots; (c) photograph of experimental enclosures in Inner Mongolia, China (photographed by Xin Zhang).

In 2010, we constructed four enclosures with galvanized iron sheets extending 1 m below and 1.4 m above the ground to prevent the movement of burrowing rodents into or out of the enclosure. Each enclosure was covered with 50 cm high wire netting with a 1 cm mesh size on all four sides and nylon netting with a 10 cm mesh size on the top to exclude avian predators (more details, see Li et al., 2016). These enclosures eliminated all emigration and immigration of rodents and excluded all predators. In early April each year, all the resident voles were removed from the enclosures before the experiment began to minimize the differences of founder voles in different enclosures. In late April of each year, 13 or 15 vole breeding pairs were introduced into each enclosure as founding populations. Each enclosure (block) was 60 m × 80 m and divided into five plots, which is, a tot attachmental of 20 replicates of vole treatments. Except that, five 10 m × 10 m blocks were established for the control treatment, each block was also divided into five plots and included 25 replicates (Figure 1).

In late June 2019, six minirhizotron tubes were installed vertically near the three gramineous plant taxa in each enclosure for non‐destructive monitoring of their roots. The total length of each tube was 40 cm, and the tubes reached a depth of 30 cm, with 10 cm above the surface. The light was restricted from the aboveground section of each tube using a black cover.

### 2.3 Vegetation survey

Vegetation was sampled in mid‐August 2019 and 2020 to avoid a pseudoreplicated result in a long‐term enclosure experiment as much as possible (Yu et al., 2020). The height, cover, density, and biomass of each plant species were measured in two randomly selected quadrats (1 m × 1 m) within each plot. After completing the plant community survey, all the living plant tissues within a randomly chosen 0.3 m × 0.3 m quadrat inside each selected quadrat were harvested by clipping the vegetation. The clipped plant material was separated according to species and oven‐dried at 55°C to constant weight to determine the aboveground biomass.

To measure the belowground biomass, two soil samples (diameter 3.5 cm, length 15 cm) were collected from each enclosure, and the roots were selected after washing them with distilled water. Live roots were selected based on their color and elasticity and measured using a ruler. Then the live roots were placed in envelopes, oven‐dried at 55°C to constant weight, and weighed.

### 2.4 Plant defense trait measurement

Fourteen aboveground and belowground traits were used to describe the plant defense response to Brandt's voles. To minimize the effects of the digging and defecation behaviors of Brandt's vole on plants, five mature plants with fully expanded and undamaged leaves were selected from each of the three species of plants in each plot for measuring plant defense traits. We measured plant height with a ruler, cut off the plant material, and brought the samples to the laboratory for refrigeration. The second and third leaves from top to bottom were selected from *L. chinensis*, *S. krylosii*, and *C. squarrosa* plants. Leaf area was measured using a YMJ‐B portable leaf area meter (Zhejiang Topu Yunnong Science and Technology Co., Ltd., Zhejiang, China). Leaf thickness was measured using a digital display vernier caliper. The leaves were immersed in a dark environment at 5–8°C for 12 h to measure the leaf saturated fresh weight. The leaves were oven‐dried for 48 h at 55°C and then weighed for leaf dry weight. They were then ground into powder, and the leaf carbon content and leaf nitrogen content were measured using a Vario El Cube elemental analyzer to calculate the leaf C:N ratio.

Digital images were captured regularly within the root tubes at two positions along the tube by using a minirhizotron digital camera system (PMT‐Root 700). The images were analyzed using Root Analysis software, which provided values for root length density, root surface area density, and average root diameter by tracing the boundaries of each root.

### 2.5 Data calculations

The importance value reflects the dominance of a specific species in a community. In this study, it was calculated as IV_i_ = (RB_*i*_ + RC_*i*_ + RH_*i*_ + RN_*i*_)/4.

$$\text{Here},{\mathrm{RB}}_{i}=\left(B_{i}/\sum_{i=1}^{n}B_{i}\right),$$

$${\mathrm{RC}}_{i}=\left(B_{i}/\sum_{i=1}^{n}C_{i}\right),$$

$${\mathrm{RH}}_{i}=\left(B_{i}/\sum_{i=1}^{n}H_{i}\right),$$

$${\mathrm{RN}}_{i}=\left(B_{i}/\sum_{i=1}^{n}N_{i}\right),$$

where B_*i*_, C_*i*_, H_*i*_, and N_*i*_ are the biomass, coverage, height, and number of species *i*, respectively, in each quadrant.

Some leaf functional traits were calculated as:

$$\text{LDMC}=\left(\text{LSFW}/\mathrm{LDW}\right)\times 100\%,$$

SLA=LA/LDW,

where LDMC, LSFW, LDW, SLA, and LA represent leaf dry matter content, leaf saturated fresh weight, leaf dry weight, specific leaf area, and leaf area, respectively.

Root functional traits were calculated from the minirhizotron data (Ma et al., 2022):

$$\mathrm{BGB}=\left(\mathrm{RLD}/\mathrm{SRL}\right)\times D,$$

$${\text{GRORL}}_{j}=\left({\mathrm{RLD}}_{j+1}-{\mathrm{RLD}}_{j}\right)/d,$$

$${\text{GRORB}}_{j}=\left({\mathrm{BGB}}_{j+1}-{\mathrm{BGB}}_{j}\right)/d,$$

where RLD is the root length density (cm/cm^3^). The specific root length (SRL, cm/g) was calculated as the total live root length divided by the total live root dry mass per soil sample, and then multiplied by the depth of the sample soil profile (D, cm) to convert RLD into the belowground biomass (BGB, g/cm^2^). GRORL_j_ is the net increase in root length per unit time (cm cm^−3^ day^−1^). RLD_*j*+1_ and RLD_*j*_ represent the length of live roots observed for the *j* + 1th and *j*th periods, respectively. *d* is the number of days between two adjacent observations. GRORB_j_ is the net increase in root biomass per unit time (g cm^−2^ day^−1^), and the calculation method is the same as for the former.

### 2.6 Data analysis

Differences in the dominance, biomass, and plant functional traits among different treatments were tested using generalized linear mixed‐effect models (GLMMs), with the presence or absence of Brandt's vole as a fixed effect and sampling year and subplot as random effects (Yu et al., 2020). The differences in the dominance, biomass, and functional traits among different species under the same treatment were compared using the same method. Species identity was treated as a fixed factor, and sampling year and subplot were treated as random factors. The Tukey HSD test was used at the 5% probability level to determine differences among different species. The “lmerTest” package was used for the linear mixed‐effects model, and Tukey's HSD test was implemented using the “multcomp” package. A structural equation model (SEM) was used to explore the pathways by which Brandt's vole affected the dominant position of *L. chinensis*, *S. krylovii*, and *C. squarrosa*. The dominance of the three gramineous plants was taken as the dependent variable, and possible pathways were considered in the initial model. A modified model was constructed by removing non‐significant pathways when the initial model did not produce an adequate fit. Data were plotted using GraphPad Prism 8 and analyzed using R 4.2.0 and IBM SPSS AMOS 24 software.

## 3 RESULTS

### 3.1 Dominance of three gramineous plant responses to Brandt's vole

**FIGURE 2:**
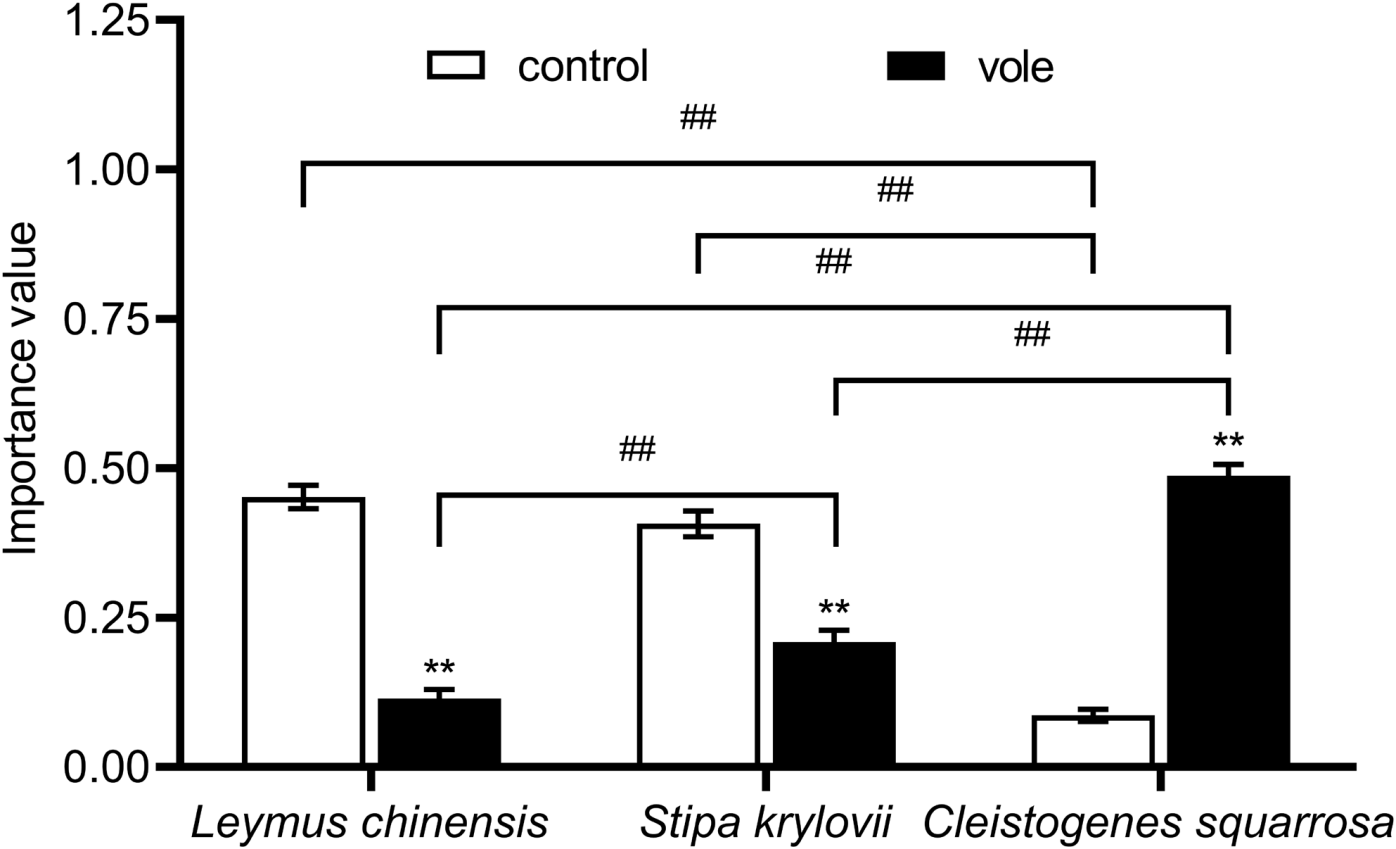
Effects of Brandt's vole and species identity on the importance value during 2019–2020 (sampling year and subplot were treated as random factors). Bars are means ± S.E.M. *p < .05, **p < .01 indicate the differences of the same plant species between two treatments; ^#^ *p* < .05, ^##^ *p* < .01 indicate the differences of two plant species in the same treatments.

The species varied considerably in dominance, and differential responses to Brandt's voles were observed (Table 1; Figure 2). *L. chinensis* was the first dominant species in this natural steppe, but feeding by Brandt's vole decreased its importance value (*F* = 109.270, *p* < .001) compared with the control group (33.7%). The importance value of *S. krylovii* also significantly decreased (19.8%; *F* = 18.554, *p* < .001). Compared with that in the control enclosure, the importance value of *C. squarrosa* significantly increased (40.1%) and it became the first dominant species in the disturbed community with Brandt's voles (*F* = 98.011, *p* < .001).

**TABLE 1:** Results (*F*‐value, *p*‐value) of generalized linear mixed‐effect models testing the effect of Brandt's vole on importance value, aboveground biomass, belowground biomass, the growth rate of root biomass, plant height, leaf saturated fresh weight, leaf dry weight, leaf dry matter content, root length density, root surface area density, average root diameter, growth rate of root length, leaf thickness, specific leaf area, leaf C:N ratio.

Variable	*Leymus chinensis*		*Stipa krylovii*		*Cleistogenes squarrosa*	
	*F*	*p*	*F*	*p*	*F*	*p*
importance value	109.270	.001	18.554	.001	98.011	.001
aboveground biomass	47.022	.001	45.935	.001	27.092	.001
belowground biomass	7.186	.012	8.113	.009	2.592	.118
the growth rate of root biomass	4.422	.044	4.070	.055	0.152	.700
plant height	1.915	.180	0.799	.381	1.127	.294
leaf saturated fresh weight	7.717	.012	0.895	.354	1.531	.228
leaf dry weight	2.616	.121	1.921	.182	0.117	.734
leaf dry matter content	8.173	.006	0.045	.835	10.732	.002
root length density	1.423	.242	7.591	.011	3.132	.087
root surface area density	0.847	.365	7.256	.013	2.201	.148
average root diameter	3.837	.059	5.566	.027	0.001	.991
growth rate of root length	0.029	.866	4.891	.037	0.973	.333
leaf thickness	4.698	.041	0.049	.826	0.255	.618
specific leaf area	3.626	.069	4.641	.042	7.434	.009
leaf C:N ratio	9.424	.005	4.916	.035	0.315	.579

### 3.2 Defense strategies of three gramineous plants in response to Brandt's vole

#### 3.2.1 Escape characteristics of the three gramineous plants

**FIGURE 3:**
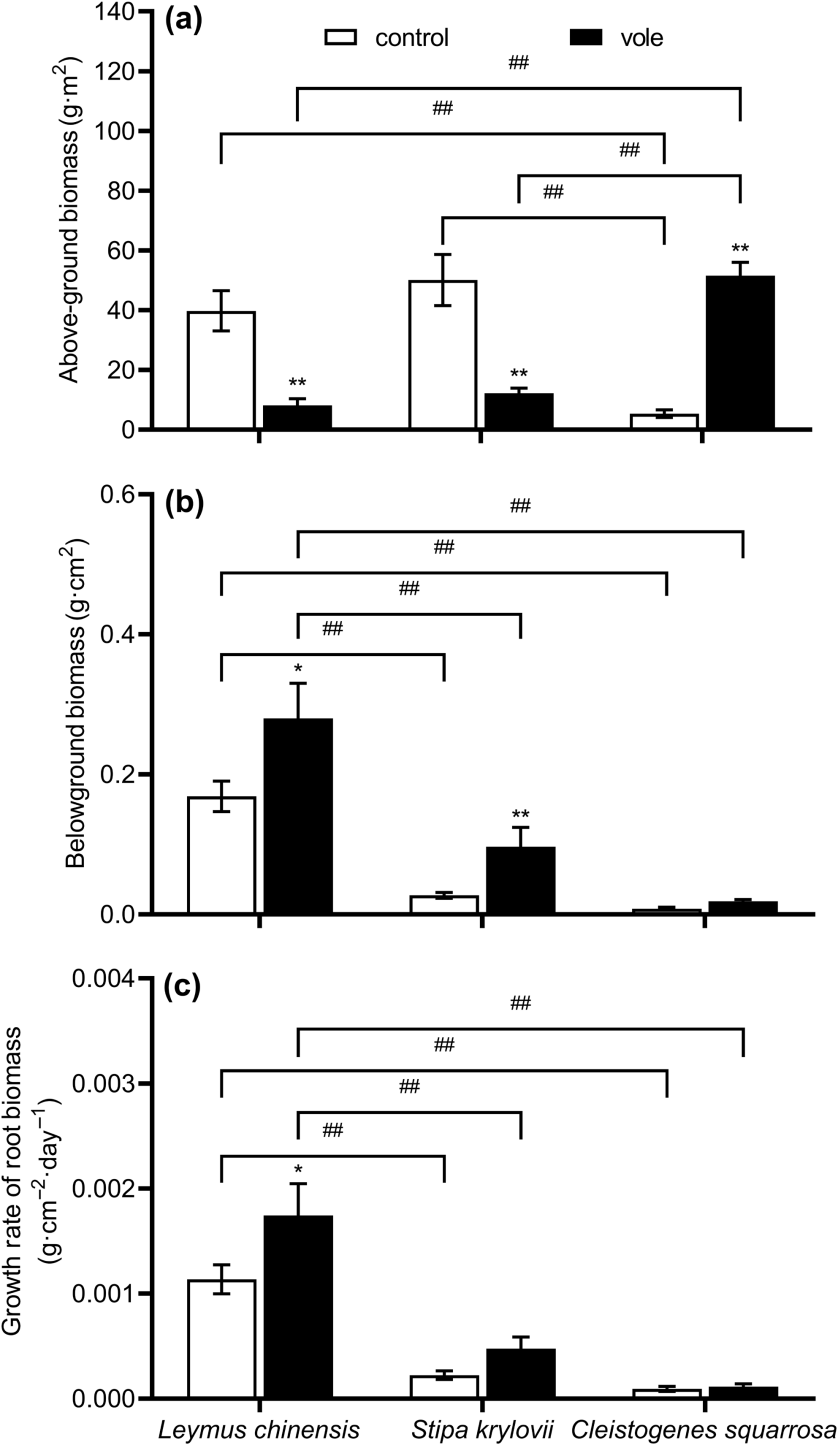
Effects of Brandt's vole on (a) aboveground biomass, (b) belowground biomass, (c) the growth rate of root biomass during 2019–2020 (sampling year and subplot were treated as random factors). Bars are means ± S.E.M. *p < .05, **p < .01 indicate the differences of the same plant species between two treatments; ^#^ *p* < .05, ^##^ *p* < .01 indicate the differences of two plant species in the same treatments.

After 10 years of continuous disturbance by Brandt's vole, the aboveground biomass of *L. chinensis* (*F* = 47.022, *p* < .001) and *S. krylovii* (*F* = 45.935, *p* < .001) significantly decreased. Meanwhile, the aboveground biomass of *C. squarrosa* (*F* = 27.092, *p* < .001) significantly increased, and *C. squarrosa* (*p* < .001) had the highest aboveground biomass in the vole‐treated enclosure (Table 1; Figure 3). The belowground biomass of *L. chinensis* (*F* = 7.186, *p* = .012) and *S. krylovii* (*F* = 8.113, *p* = .009) significantly increased in Brandt's vole disturbed enclosures, whereas the belowground biomass of *C. squarrosa* (*F* = 2.592, *p* = .118) showed no significant differences between treatments. The growth rate of root biomass of *L. chinensis* (*F* = 4.422, *p* = .044) was significantly higher in the vole enclosures than in the control group, and we found no significant differences in the growth rate of root biomass of *C. squarrosa* (*F* = 0.152, *p* = .700) for the different groups, while the growth rate of root biomass of *S. krylovii* (*F* = 4.070, *p* = .055) showed an increasing trend. The belowground biomass (*p* < .001) and the growth rate of root biomass (*p* < .001) of *L. chinensis* were significantly higher than that of *S. krylovii* and *C. squarrosa* under both treatments (Figure 3).

#### 3.2.2 Tolerance characteristics of three gramineous plants

**FIGURE 4:**
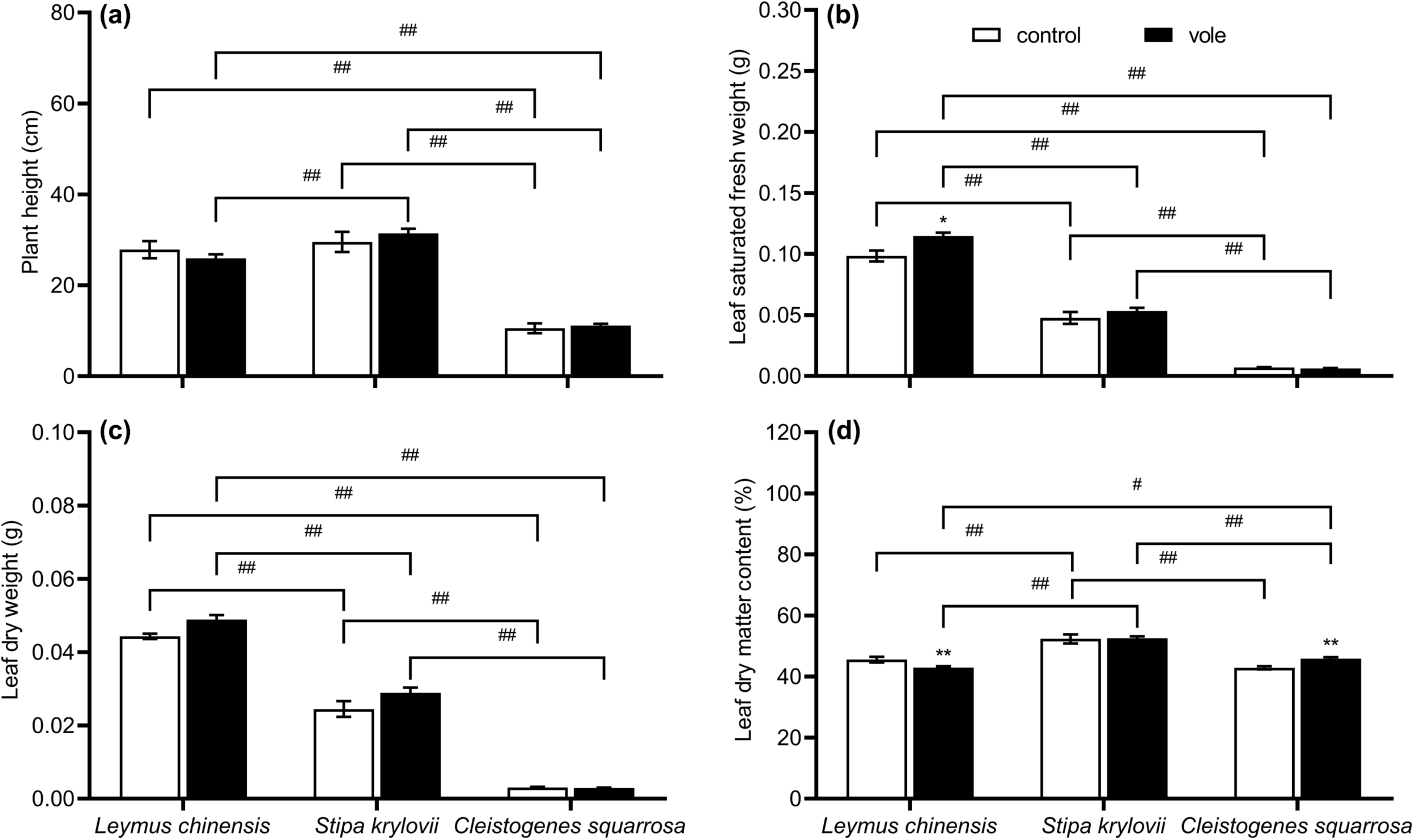
Effects of Brandt's vole on plant aboveground tolerance traits (a) plant height, (b) leaf saturated fresh weight, (c) leaf dry weight, (d) leaf dry matter content during 2019–2020 (sampling year and subplot were treated as random factors). Bars are means ± S.E.M. *p < .05, **p < .01 indicate the differences of the same plant species between two treatments; ^#^ *p* < .05, ^##^ *p* < .01 indicate the differences of two plant species in the same treatments.

The performance of plant height, leaf saturated fresh weight, and leaf dry weight was dependent on species identity, as shown in Figure 4. Compared to the control treatment, Brandt's vole increased the leaf saturated fresh weight (*F* = 7.717, *p* = .012) of *L. chinensis* and leaf dry matter content (*F* = 10.732, *p* = .002) of *C. squarrosa*, whereas the leaf dry matter content (*F* = 8.173, *p* = .006) of *L. chinensis* significantly decreased (Table 1; Figure 4c,e). There were no significant differences between treatments in plant height, and leaf dry weight of three gramineous, respectively (*p* > .05; Table 1; Figure 4a,b,d).

**FIGURE 5:**
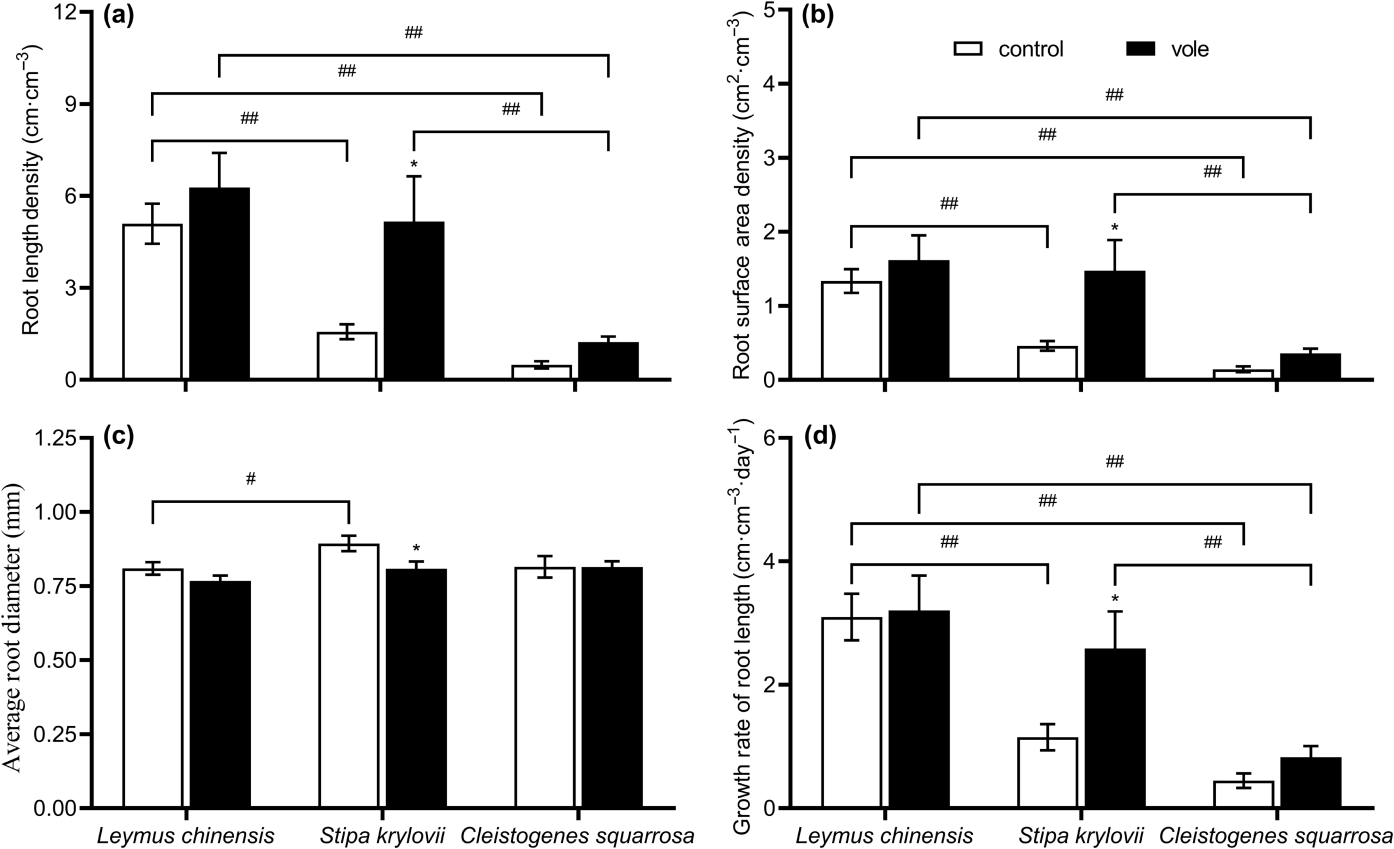
Effects of Brandt's vole on plant belowground tolerance traits (a) root length density, (b) root surface area density, (c) average root diameter, (d) growth rate of root length during 2019–2020 (sampling year and subplot were treated as random factors). Bars are means ± S.E.M. *p < .05, **p < .01 indicate the differences of the same plant species between two treatments; ^#^ *p* < .05, ^##^ *p* < .01 indicate the differences of two plant species in the same treatments.

In Brandt's vole treatment, the root length density (*F* = 7.591, *p* = .011), root surface area density (*F* = 7.256, *p* = .013), and growth rate of root length (*F* = 4.891, *p* = .037) of *S. krylovii* significantly increased, while the average root diameter (*F* = 5.566, *p* = .027) was significantly decreased (Table 1; Figure 5a–e). For *L. chinensis* and *C. squarrosa* respectively, the root length density, root surface area density, average root diameter, and growth rate of root length showed no significant difference between the two treatments (*p* > .05; Table 1; Figure 5a–e). The performance of root length density, root surface area density, average root diameter, and growth rate of root length was dependent on species identity (Figure 5).

#### 3.2.3 Resistance characteristics of three gramineous plants

**FIGURE 6:**
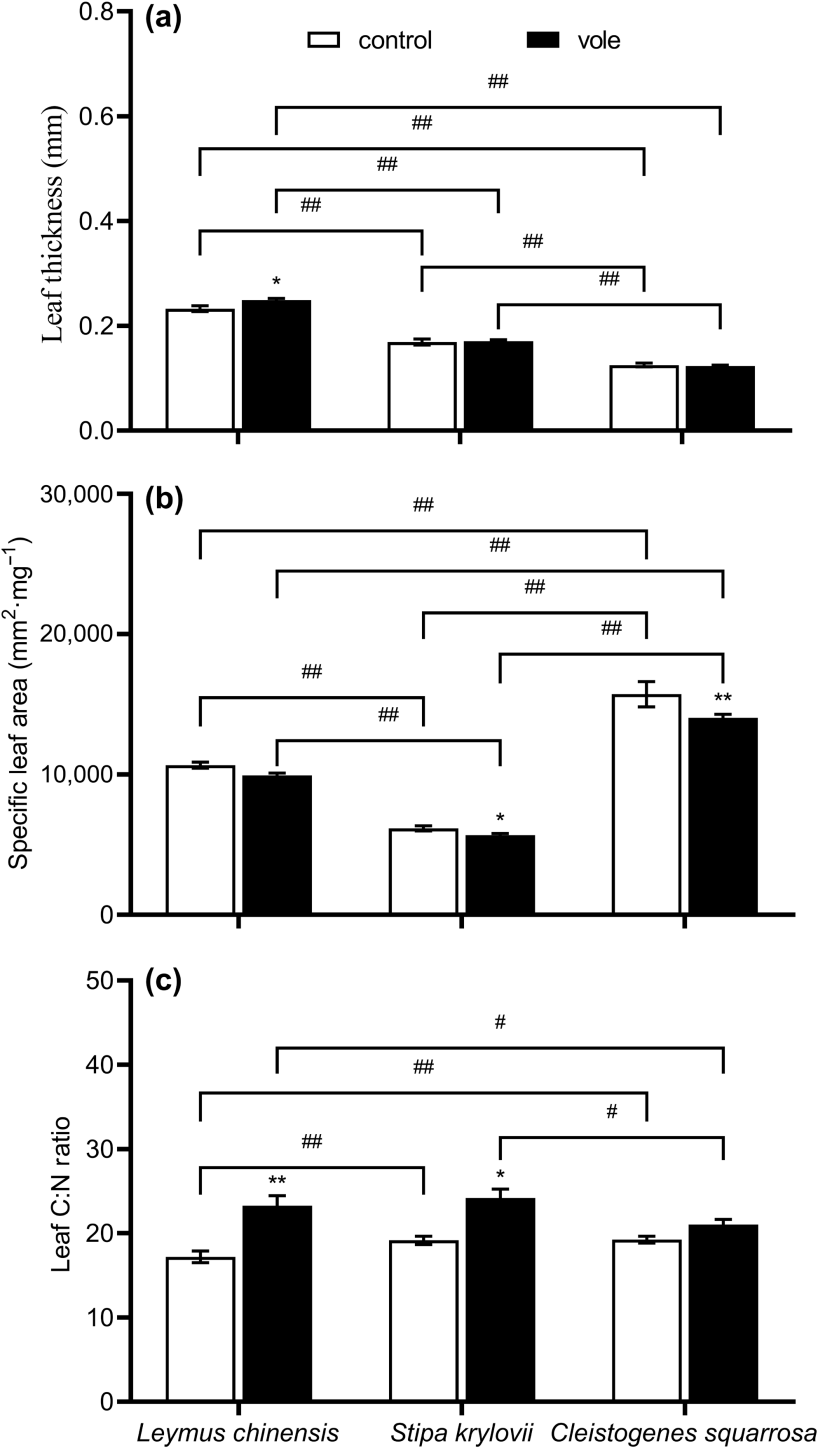
Effects of Brandt's vole on plant resistance traits (a) leaf thickness, (b) specific leaf area, (c) leaf C:N ratio during 2019–2020 (sampling year and subplot were treated as random factors). Bars are means ± S.E.M. *p < .05, **p < .01 indicate the differences of the same plant species between two treatments; ^#^ *p* < .05, ^##^ *p* < .01 indicate the differences of two plant species in the same treatments.

Long‐term disturbance by Brandt's voles resulted in an increase in leaf thickness (*F* = 4.698, *p* = .041) and leaf C:N ratio (*F* = 9.424, *p* = .005) in *L. chinensis* (Table 1; Figure 6a,c). Meanwhile, the specific leaf area (*F* = 4.641, *p* = .042) of *S. krylovii* significantly decreased under the treatment with Brandt's voles, while the leaf C:N ratio (*F* = 4.916, *p* = .035) significantly increased (Table 1; Figure 6b,c). Compared to the control treatment, Brandt's vole treatment also decreased the specific leaf area (*F* = 7.434, *p* = .009) of *C. squarrosa* (Table 1; Figure 6b). There were no significant differences in the specific leaf area of *L. chinensis*, leaf thickness of *S. krylovii*, and leaf thickness and leaf C:N ratio of *C. squarrosa* between the different treatments (*p* > .05; Table 1; Figure 6b,c). Among the three gramineous species, the leaf thickness, specific leaf area, and leaf C:N ratio characteristics were species‐specific (Figure 6).

### 3.3 Relationships between species importance value and plant functional traits

**FIGURE 7:**
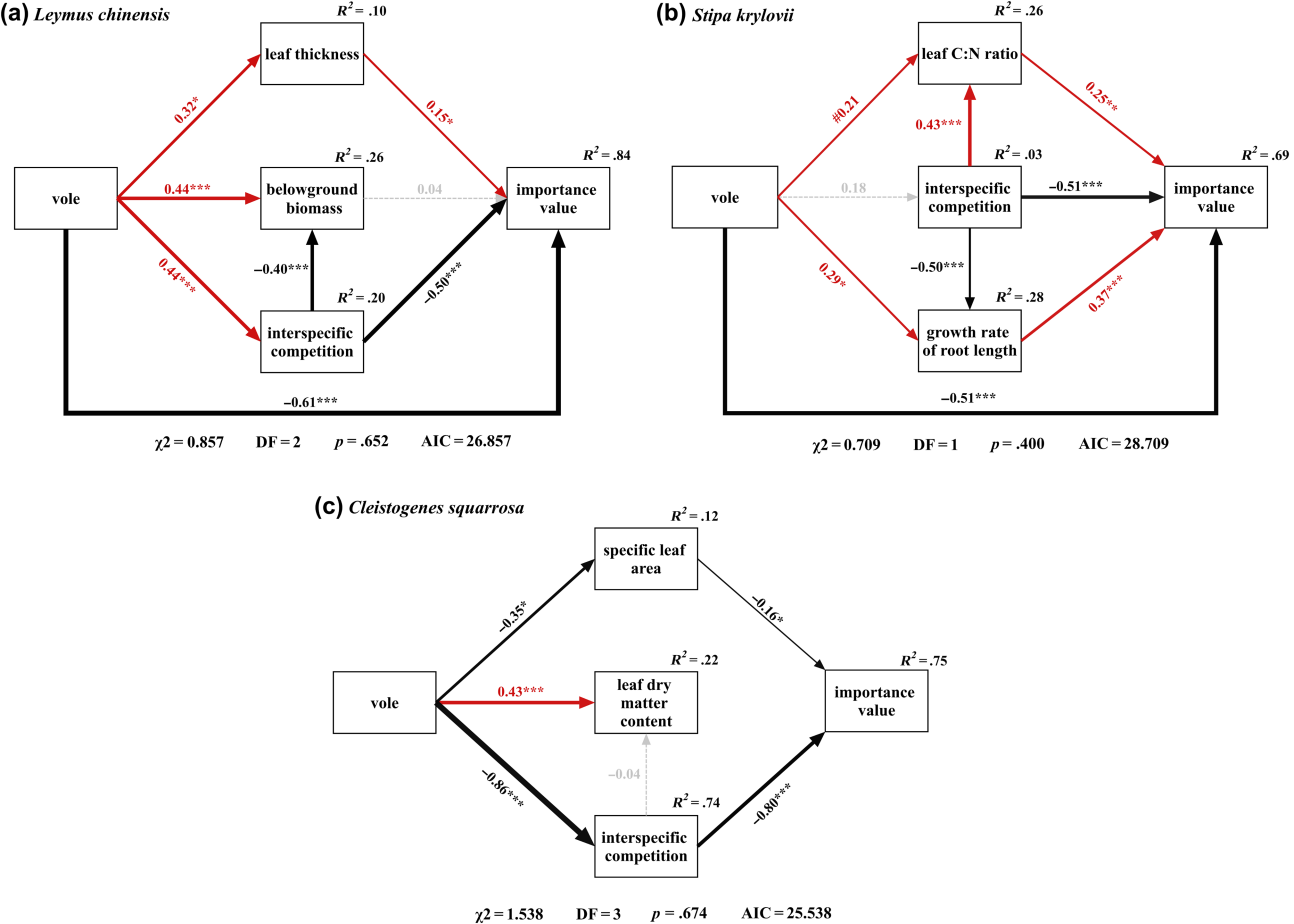
The final structural equation model describing the effects of Brandt's vole on the importance value of (a) *Leymus chinensis*, (b) *Stipa krylovii*, and (c) *Cleistogenes squarrosa* through plant functional traits. Red and black arrows represent positive and negative pathways (*p* < .10), and gray dashed arrows indicate nonsignificant pathways (*p* > .10). Standardized path coefficients are shown on the pathways. ^#^ *p* < .10, *p < .05, **p < .01, ***p < .001. *R* ^2^ represents the proportion of variance explained for each dependent variable in the model. The adequacy of model fit was evaluated using the model chi‐square (χ^2^) and its associated *p* values, Akaike information criterion (AIC) also represents a fit index examined.

The final SEM showed that Brandt's vole disturbance directly or indirectly affected the importance value of the three species (Figure 7). As shown in Figure 7a, the negative effect of Brandt's vole on *L. chinensis* includes direct and indirect inhibition of the dominant position of *L. chinensis* by increasing interspecific competition (the importance value of *S. krylovii* + *C. squarrosa*). Brandt's vole had a positive effect on the leaf thickness of *L. chinensis*, and the leaf thickness is positive with dominance. Feeding by Brandt's vole directly reduced the dominance of *S. krylovii*. The negative effect of interspecific competition (the importance value of *L. chinensis* + *C. squarrosa*) on the importance value of *S. krylovii* could also be observed. Feeding by Brandt's voles significantly increased the leaf C:N ratio and growth rate of root length of *S. krylovii*, and both traits were positively correlated with dominance (Figure 7b). In contrast, our SEM provided evidence that Brandt's vole did not directly affect the dominance of *C. squarrosa*. However, the positive indirect effect of Brandt's vole on the importance value was predominantly due to its negative effect on the specific leaf area and interspecific competition (the importance value of *L. chinensis* + *S. krylovii*) of *C. squarrosa* (Figure 7c).

## 4 DISCUSSION

After 10 years of feeding, digging, and other activities by Brandt's vole, we found that the importance value of *L. chinensis* in vole enclosures decreased significantly by 33.7%, from the first dominant position to the third. The importance value of *S. krylovii* was decreased by 19.8%, but it remained in the second dominant position. Meanwhile, the dominance of *C. squarrosa* increased by 40.1%, and it became the first dominant species (Figure 2). Small herbivores with small gastrointestinal volumes should be more selective of plants than ungulates are and thus impose consistent suppression of preferred forage species and release of nonpreferred species, eventually creating plant communities dominated by species that they do not like to eat (Howe et al., 2006, 2010), which is consistent with our results that the plant community was dominated by *C. squarrosa* after a 10‐year experiment (Figure 2). Because in our research region, *L. chinensis* is the most preferred, and *C. squarrosa* is the less preferred taxon by Brandt's voles, which is due to the lower protein content of *C. squarrosa* (Hou et al., 2016). In addition, we used SEM to test the effect of species relationships on plant dominance. We found that the inhibition effect of other plants on a target plant can have a negative impact on its dominance, and this inhibition effect is more reflected in reducing the underground input of the target plant (Figure 7). Colom and Baucom (2020) and Ballhorn et al. (2014) also found that competition will change the root of plants and reduce the underground biomass of plants. In the presence of both competition and herbivores, plants with high defense effectiveness will show better adaptability. On the contrary, plants with low defense efficiency will lose their dominant position, like *L. chinensis*. Although *L. chinensis* lost its first dominant position in the plant community during continuous foraging by Brandt's vole, it still occupied a certain proportion of the plant community, which may be related to its defense strategy.

Plants can defend against herbivores by changing their resistance traits. Such as Prado et al. (2014) and Chen, Moles, et al. (2021) found that the leaf thickness of plants and the leaf C:N ratio significantly increase under continuous grazing. Specific leaf area is an alternative measure of leaf thickness and toughness; under grazing conditions, specific leaf area was found to be lower than in un‐grazed forests (Carlucci et al., 2012). In this study, we also observed Brandt's voles caused an increase in the leaf thickness of *L. chinensis* and leaf C:N ratio of *S. krylovii*, and decreased the specific leaf area of *C. squarrosa*, and all three resistance traits had positive effects on plant dominant (Figure 7). Therefore, resistance was an effective defense strategy against Brandt's voles to some extent. Notably, the resistance traits in plant species are extremely variable along ontogeny (Kariñho‐Betancourt et al., 2015). In this case, the efficiency of resistance defense of different plants for a particular herbivore should be different; herbivores may have a preference for feeding on plants with low resistance defense efficiency. Our results showed that all the resistance traits were the highest in *L. chinensis* and lowest in *C. squarrosa* in vole enclosures (Figure 6). However, in a previous study in our enclosures, Hou et al. (2016) showed in cage experiments that the preference ranking of the three plants by Brandt's voles was *L. chinensis* > *S. krylovii* > *C. squarrosa*. These seemingly conflicting results may indicate that Brandt's vole has currently overcome the potential arms race with *L. chinensis* in terms of the coevolution of defenses and counter‐defenses. Like classical coevolutionary theory suggests that herbivores have developed physiological and behavioral adaptations to the plant resistance defenses, which enable them to feed on unpalatable but nutritionally rich plants without obvious negative effects (Dawkins & Krebs, 1979; Wittstock et al., 2004). If this is the case, we propose that resistance traits can serve as indicators of plant defense input but may not be suitable as indicators of preference of herbivores.

Tolerance defense is a physiological defense strategy used by plants to increase tolerance to herbivores, predominantly through compensatory growth and improved photosynthetic efficiency (Barton, 2013). In our study, Brandt's vole significantly increased leaf saturated fresh weight and decreased leaf dry matter content of *L. chinensis* (Figure 4). Firn et al. (2012) found lovegrass leaves decreased in leaf dry matter content under grazing and showed lower capacity to conserve resources but with rapid growth. Thus, the decrease in leaf dry matter content of *L. chinensis* is better at growth rapidly; it enhanced its tolerance defense effect against Brandt's voles. Leaf saturated fresh weight is an important growth characteristic and is directly linked to water content and efficient accumulation of dry matter in leaves (Gross et al., 2007). The increase in leaf saturated fresh weight may have somewhat improved the photosynthetic and growth efficiency of *L. chinensis*. This could have increased its compensatory growth in response to the negative effects of feeding activities. However, due to the high nutrient content of *L. chinensis*, it is subjected to strong feeding pressure (Hou et al., 2016), and the biomass consumed during the feeding process cannot be effectively supplemented by rapid compensatory growth. This may have caused the SEM results to show that the tolerance defense strategy had no significant effect on the importance of *L. chinensis*. The leaf dry matter content of *C. squarrosa* significantly increased after feeding by Brandt's voles, increasing resource conservation (Smart et al., 2017). We speculate that this result may indicate that, under less feeding pressure, the *C. squarrosa* did not adopt the tolerant defense strategy of rapid growth. Erb et al. (2009) and Moreira et al. (2012) showed that plant compensation for aboveground herbivore feeding often involves increased investment allocation to belowground tissues to improve root water and nutrient uptake. We found similar results that root length density, root surface area density, and growth rate of root length of *S. krylovii* significantly increased in the presence of Brandt's voles (Figure 5), which was conducive to improving the plant's absorption efficiency of soil nutrients and water, and showed a significant positive correlation with dominant position (Chen, Xiong, & Cheng, 2021).

Liu et al. (2019) found plants are more likely to invest in escape traits than tolerance traits under severe herbivore stress. For example, Joly et al. (2018) considered an increase in belowground biomass and a decrease in aboveground biomass were adaptive strategies by plants to escape herbivore intake. This is consistent with our results that two gramineous species have used escape strategies to deal with feeding. In this study, Brandt's vole significantly reduced the aboveground biomass of *L. chinensis* and *S. krylovii* and significantly increased their belowground biomass and the growth rate of root biomass of *L. chinensis*. Given that roots are not directly feeding organs, increasing their distribution can allow plants to escape from herbivores feeding, reduce energy consumption, and retain more energy resources belowground. This allows future rapid growth to recapture the dominant position when the living environment improves (Liu et al., 2019; Wang et al., 2016). Hence, although the increase in belowground investment did not significantly improve the current dominance of *L. chinensis* (Figure 7a), the benefits of this change may be observed in the future when Brandt's vole population decreases. For *C. squarrosa*, which suffers from low feeding pressure, it was not necessary to increase underground input to escape the feeding of Brandt's voles, but more importantly to occupy more aboveground space, light, and other survival resources by increasing the aboveground biomass.

## 5 CONCLUSION

We found that all three grass species exhibited an enhanced resistance response to feeding by small rodents, thereby positively influencing their dominance within the ecosystem. However, owing to selective feeding, Brandt's voles affected the dominance position of the three species in different ways, resulting in interspecific differences in their defense strategies. These defense strategies had differential effects on fitness, which changed the interspecific competition pattern, affected the dominant position of the plants, and drove changes in the species composition of the plant communities. And comparing the defense strategies of different plant species will provide possible insights into macroevolutionary patterns of defense characteristics.

## AUTHOR CONTRIBUTIONS

**Yanjin Xie:** Data curation (equal); formal analysis (equal); investigation (equal); methodology (equal); visualization (equal); writing – original draft (equal). **Jiading Zhang:** Investigation (equal). **Yongle Hua:** Investigation (equal). **Wanhong Wei:** Conceptualization (equal); project administration (equal). **Baofa Yin:** Funding acquisition (equal); project administration (equal); writing – review and editing (equal).

## CONFLICT OF INTEREST STATEMENT

The authors declare no conflicts of interest.

## Data Availability

The data that support the findings of this study are available from the corresponding author upon reasonable request. Relevant data of the paper have been uploaded to the Dryad repository, private for Peer Review. Doi: 10.5061/dryad.qz612jmmk.

## References

[ece370086-bib-0001] Agrawal, A. A. , & Fishbein, M. (2006). Plant defense syndromes. Ecology, 87(7 Suppl), S132–S149. 16922309 10.1890/0012-9658(2006)87[132:pds]2.0.co;2

[ece370086-bib-0002] Avirmed, D. , Batsaikhan, N. , & Tinnin, D. (2016). Lasiopodomys brandtii. The IUCN Red List of Threatened Species, e.T11340A115101423.

[ece370086-bib-0003] Ballhorn, D. J. , Godschalx, A. L. , Smart, S. M. , Kautz, S. , & Schädler, M. (2014). Chemical defense lowers plant competitiveness. Oecologia, 176(3), 811–824. 25173086 10.1007/s00442-014-3036-1

[ece370086-bib-0004] Barton, K. E. (2013). Ontogenetic patterns in the mechanisms of tolerance to herbivory in Plantago. Annals of Botany, 112(4), 711–720. 23589631 10.1093/aob/mct083 PMC3736769

[ece370086-bib-0005] Bergmann, J. , Ryo, M. , Prati, D. , Hempel, S. , & Rillig, M. C. (2017). Root traits are more than analogues of leaf traits: The case for diaspore mass. The New Phytologist, 216(4), 1130–1139. 28895147 10.1111/nph.14748

[ece370086-bib-0006] Boege, K. (2010). Induced responses to competition and herbivory: Natural selection on multi‐trait phenotypic plasticity. Ecology, 91(9), 2628–2637. 20957957 10.1890/09-0543.1

[ece370086-bib-0007] Capó, M. , Roig‐Oliver, M. , Cardona, C. , Cursach, J. , Bartolomé, J. , Rita, J. , & Baraza, E. (2021). Historic exposure to herbivores, not constitutive traits, explains plant tolerance to herbivory in the case of two Medicago species (Fabaceae). Plant Science, 307, 110890. 33902851 10.1016/j.plantsci.2021.110890

[ece370086-bib-0008] Carlucci, M. B. , Streit, H. , Duarte, L. D. S. , & Pillar, V. D. (2012). Individual‐based trait analyses reveal assembly patterns in tree sapling communities. Journal of Vegetation Science, 23(1), 176–186.

[ece370086-bib-0009] Chen, R. , Xiong, X. P. , & Cheng, W. H. (2021). Root characteristics of spring wheat under drip irrigation and their relationship with aboveground biomass and yield. Scientific Reports, 11(1), 4913. 33649480 10.1038/s41598-021-84208-7 PMC7921688

[ece370086-bib-0010] Chen, Y. D. , Moles, A. , Bu, Z. J. , Zhang, M. M. , Wang, Z. C. , & Zhao, H. Y. (2021). Induced defense and its cost in two bryophyte species. American Journal of Botany, 108(5), 777–787. 33948954 10.1002/ajb2.1654

[ece370086-bib-0011] Colom, S. M. , & Baucom, R. S. (2020). Belowground competition can influence the evolution of root traits. The American Naturalist, 195(4), 577–590. 10.1086/707597 32216668

[ece370086-bib-0012] Cui, C. , Xie, Y. J. , Hua, Y. L. , Yang, S. M. , Yin, B. F. , & Wei, W. H. (2020). Brandt's vole (*Lasiopodomys brandtii*) affects its habitat quality by altering plant community composition. Biologia, 75(8), 1097–1104.

[ece370086-bib-0013] Dai, L. , Guo, X. , Ke, X. , Zhang, F. , Li, Y. , Peng, C. , Shu, K. , Li, Q. , Lin, L. , Cao, G. , & Du, Y. (2019). Moderate grazing promotes the root biomass in Kobresia meadow on the northern Qinghai‐Tibet plateau. Ecology and Evolution, 9(16), 9395–9406. 31463030 10.1002/ece3.5494 PMC6706204

[ece370086-bib-0014] Danet, A. , Kéfi, S. , Meneses, R. I. , & Anthelme, F. (2017). Nurse species and indirect facilitation through grazing drive plant community functional traits in tropical alpine peatlands. Ecology and Evolution, 7(24), 11265–11276. 29299299 10.1002/ece3.3537 PMC5743694

[ece370086-bib-0015] Dawkins, R. , & Krebs, J. R. (1979). Arms races between and within species. Proceedings of the Royal Society of London. Series B, Biological Sciences, 205(1161), 489–511. 42057 10.1098/rspb.1979.0081

[ece370086-bib-0016] de Bobadilla, M. F. , Vitiello, A. , Erb, M. , & Poelman, E. H. (2022). Plant defense strategies against attack by multiple herbivores. Trends in Plant Science, 27(6), 528–535. 35027280 10.1016/j.tplants.2021.12.010

[ece370086-bib-0017] Erb, M. , Lenk, C. , Degenhardt, J. , & Turlings, T. C. (2009). The underestimated role of roots in defense against leaf attackers. Trends in Plant Science, 14(12), 653–659. 19736036 10.1016/j.tplants.2009.08.006

[ece370086-bib-0018] Firn, J. , Prober, S. M. , & Buckley, Y. M. (2012). Plastic traits of an exotic grass contribute to its abundance but are not always favourable. PLoS One, 7(4), e35870. 22536448 10.1371/journal.pone.0035870 PMC3335023

[ece370086-bib-0019] Gross, N. , Suding, K. N. , & Lavorel, S. (2007). Leaf dry matter content and lateral spread predict response to land use change for six subalpine grassland species. Journal of Vegetation Science, 18(2), 289–300.

[ece370086-bib-0020] He, Q. , Jiang, K. , Hou, W. , Zhao, Y. , Sun, X. , Wang, L. , Zou, Y. , Zhu, Z. , & Zhang, H. (2021). Grazing alters species relative abundance by affecting plant functional traits in a Tibetan subalpine meadow. Ecology and Evolution, 11(16), 11028–11037. 34429900 10.1002/ece3.7891 PMC8366865

[ece370086-bib-0021] Hou, X. L. , Li, G. L. , Wan, X. R. , & Zhang, Z. B. (2016). Influence of sheep grazing on major food selection by Brandt's voles (*Lasiopodpmys brandtii*). Acta Ecologica Sinica, 36(2), 152–157. (in Chinese).

[ece370086-bib-0022] Howe, H. F. , Brown, J. S. , & Zorn‐Arnold, B. (2010). A rodent plague on prairie diversity. Ecology Letters, 5(1), 30–36.

[ece370086-bib-0023] Howe, H. F. , Zorn‐Arnold, B. , Sullivan, A. , & Brown, J. S. (2006). Massive and distinctive effects of meadow voles on grassland vegetation. Ecology, 12, 87. 10.1890/0012-9658(2006)87[3007:madeom]2.0.co;2 17249225

[ece370086-bib-0024] Hua, Y. L. , Xie, Y. J. , Yin, B. F. , & Wei, W. H. (2022). Trophic niche of Brandt's vole (*Lasiopodomys brandtii*) and sheep (*Ovis aries*) in the Inner Mongolia grassland. Acta Ecologica Sinica, 42(21), 8618–8627. (in Chinese).

[ece370086-bib-0025] Ji, G. , Li, B. , Yin, H. , Liu, G. , Yuan, Y. , & Cui, G. (2021). Non‐utilization is not the best way to manage lowland meadows in Hulun Buir. Frontiers in Plant Science, 12, 704511. 34335668 10.3389/fpls.2021.704511 PMC8322850

[ece370086-bib-0026] Jia, S. , Wang, X. , Yuan, Z. , Lin, F. , Ye, J. , Hao, Z. , & Luskin, M. S. (2018). Global signal of top‐down control of terrestrial plant communities by herbivores. Proceedings of the National Academy of Sciences of the United States of America, 115(24), 6237–6242. 29848630 10.1073/pnas.1707984115 PMC6004463

[ece370086-bib-0027] Jiang, S. , Zhang, J. , Tang, Y. , Li, Z. , Liu, H. , Wang, L. , Wu, Y. , & Liang, C. (2023). Plant functional traits and biodiversity can reveal the response of ecosystem functions to grazing. The Science of the Total Environment, 899, 165636. 37487897 10.1016/j.scitotenv.2023.165636

[ece370086-bib-0028] Joly, F. , Sabatier, R. , & Hubert, B. (2018). Modelling interacting plant and livestock renewal dynamics helps disentangle equilibrium and nonequilibrium aspects in a Mongolian pastoral system. Science of the Total Environment, 625, 1390–1404. 29996436 10.1016/j.scitotenv.2017.12.215

[ece370086-bib-0029] Kariñho‐Betancourt, E. , Agrawal, A. A. , Halitschke, R. , & Núñez‐Farfán, J. (2015). Phylogenetic correlations among chemical and physical plant defenses change with ontogeny. New Phytologist, 206(2), 796–806. 25652325 10.1111/nph.13300

[ece370086-bib-0030] Klumpp, K. , Sébastien, F. , Eléonore, A. , Roux, X. L. , Gleixner, G. , & Soussana, J. F. (2009). Grazing triggers soil carbon loss by altering plant roots and their control on soil microbial community. Journal of Ecology, 97(5), 876–885.

[ece370086-bib-0031] Koch, K. G. , Chapman, K. , Louis, J. , Heng‐Moss, T. , & Sarath, G. (2016). Plant tolerance: A unique approach to control hemipteran pests. Frontiers in Plant Science, 7, 1363. 27679643 10.3389/fpls.2016.01363 PMC5020058

[ece370086-bib-0033] Li, G. , Hou, X. , Wan, X. , & Zhang, Z. (2016). Sheep grazing causes shift in sex ratio and cohort structure of Brandt's vole: Implication of their adaptation to food shortage. Integrative Zoology, 11(1), 76–84. 26331731 10.1111/1749-4877.12163

[ece370086-bib-0034] Li, W. , Knops, J. M. H. , Zhou, X. , Jin, H. , Xiang, Z. , Ka Zhuo, C. , Jin, X. , Zhou, H. , & Dong, S. (2023). Anchoring grassland sustainability with a nature‐based small burrowing mammal control strategy. The Journal of Animal Ecology, 92(7), 1345–1356. 37211647 10.1111/1365-2656.13938

[ece370086-bib-0035] Li, W. , Xu, F. , Zheng, S. , Taube, F. , & Bai, Y. (2017). Patterns and thresholds of grazing‐induced changes in community structure and ecosystem functioning: Species‐level responses and the critical role of species traits. Journal of Applied Ecology, 54(3), 963–975.

[ece370086-bib-0036] Lind, E. M. , Borer, E. , Seabloom, E. , Adler, P. , Bakker, J. D. , Blumenthal, D. M. , Crawley, M. , Davies, K. , Firn, J. , Gruner, D. S. , Harpole, W. S. , Hautier, Y. , Hillebrand, H. , Knops, J. , Melbourne, B. , Mortensen, B. , Risch, A. C. , Schuetz, M. , Stevens, C. , & Wragg, P. D. (2013). Life‐history constraints in grassland plant species: A growth‐defence trade‐off is the norm. Ecology Letters, 16(4), 513–521. 23347060 10.1111/ele.12078

[ece370086-bib-0037] Liu, H. , Zhang, J. , & Wang, B. (2023). Contrasting seed traits of co‐existing seeds lead to a complex neighbor effect in a seed‐rodent interaction. Oecologia, 201(4), 1017–1024. 36971820 10.1007/s00442-023-05365-2

[ece370086-bib-0038] Liu, M. , Gong, J. , Li, Y. , Li, X. , Yang, B. , Zhang, Z. , Yang, L. , & Hou, X. (2019). Growth‐defense trade‐off regulated by hormones in grass plants growing under different grazing intensities. Physiologia Plantarum, 166(2), 553–569. 30091152 10.1111/ppl.12802

[ece370086-bib-0039] Loayza, A. P. , Luna, C. A. , & Calviño‐Cancela, M. (2020). Predators and dispersers: Context‐dependent outcomes of the interactions between rodents and a megafaunal fruit plant. Scientific Reports, 10(1), 6106. 32269241 10.1038/s41598-020-62704-6 PMC7142068

[ece370086-bib-0040] Ma, T. , Parker, T. , Unger, S. , Gewirtzman, J. , Fetcher, N. , Moody, M. L. , & Tang, J. (2022). Responses of root phenology in ecotypes of *Eriophorum vaginatum* to transplantation and warming in the Arctic. Science of the Total Environment, 805, 149926. 34543789 10.1016/j.scitotenv.2021.149926

[ece370086-bib-0041] Moreira, X. , Zas, R. , & Sampedro, L. (2012). Genetic variation and phenotypic plasticity of nutrient re‐allocation and increased fine root production as putative tolerance mechanisms inducible by methyl‐jasmonate in pine trees. Journal of Ecology, 100(3), 810–820.

[ece370086-bib-0042] Parra, S. A. , Thébault, E. , Fontaine, C. , & Dakos, V. (2022). Interaction fidelity is less common than expected in plant‐pollinator communities. The Journal of Animal Ecology, 91(9), 1842–1854. 35704282 10.1111/1365-2656.13762

[ece370086-bib-0043] Pérez‐Harguindeguy, N. , Díaz, S. , Garnier, E. , Lavorel, S. , Poorter, H. , Jaureguiberry, P. , Bret‐Harte, M. S. , Cornwell, W. K. , Craine, J. M. , Gurvich, D. E. , Urcelay, C. , Veneklaas, E. J. , Reich, P. B. , Poorter, L. , Wright, I. J. , Ray, P. , Enrico, L. , Pausas, J. G. , de Vos, A. C. , … Cornelissen, J. H. C. (2013). New handbook for standardised measurement of plant functional traits worldwide. Australian Journal of Botany, 61(3), 167–234.

[ece370086-bib-0044] Prado, A. , Sierra, A. , Windsor, D. , & Bede, J. C. (2014). Leaf traits and herbivory levels in a tropical gymnosperm, *Zamia stevensonii* (Zamiaceae). American Journal of Botany, 101(3), 437–447. 24638164 10.3732/ajb.1300337

[ece370086-bib-0045] Rhodes, A. C. , Larsen, R. T. , Maxwell, J. D. , & St Clair, S. B. (2018). Temporal patterns of ungulate herbivory and phenology of aspen regeneration and defense. Oecologia, 188(3), 707–719. 30242473 10.1007/s00442-018-4253-9

[ece370086-bib-0046] Salgado‐Luarte, C. , González‐Teuber, M. , Madriaza, K. , & Gianoli, E. (2022). Trade‐off between plant resistance and tolerance to herbivory: Mechanical defenses outweigh chemical defenses. Ecology, 104(1), e3860. 36047784 10.1002/ecy.3860

[ece370086-bib-0047] Sharp Bowman, T. R. , McMillan, B. R. , & St Clair, S. B. (2017). Rodent herbivory differentially affects mortality rates of 14 native plant species with contrasting life history and growth form traits. Oecologia, 185(3), 465–473. 28887653 10.1007/s00442-017-3944-y

[ece370086-bib-0048] Smart, M. S. , Glanville, C. H. , Blanes, C. M. D. , Mercado, L. M. , Emmett, B. A. , Jones, D. L. , Cosby, B. J. , Marrs, R. H. , Butler, A. , Marshall, M. R. , Reinsch, S. , Herrero‐Jáuregui, S. , & Hodgson, J. G. (2017). Leaf dry matter content is better at predicting above‐ground net primary production than specific leaf area. Functional Ecology, 31(6), 1336–1344.

[ece370086-bib-0049] Smith, S. W. , Woodin, S. J. , Pakeman, R. J. , Johnson, D. , & van der Wal, R. (2014). Root traits predict decomposition across a landscape‐scale grazing experiment. New Phytologist, 203(3), 851–862. 24841886 10.1111/nph.12845 PMC4260134

[ece370086-bib-0050] Wang, B. , Li, P. P. , Huang, C. H. , Liu, G. B. , & Yang, Y. F. (2021). Effects of root morphological traits on soil detachment for ten herbaceous species in the loess plateau. Science of the Total Environment, 754, 142304. 33254931 10.1016/j.scitotenv.2020.142304

[ece370086-bib-0051] Wang, Z. , Hu, J. , Li, X. L. , Ala, M. , Ding, Y. , Hou, X. Y. , & Yu, H. (2016). Effects of different utilization methods on biomass of dominant plants in typical steppe. Acta Prataculturae Sinica, 25(6), 185–189. (in Chinese).

[ece370086-bib-0052] Wari, D. , Aboshi, T. , Shinya, T. , & Galis, I. (2022). Integrated view of plant metabolic defense with particular focus on chewing herbivores. Journal of Integrative Plant Biology, 64(2), 449–475. 34914192 10.1111/jipb.13204

[ece370086-bib-0053] Wittstock, U. , Agerbirk, N. , Stauber, E. J. , Olsen, C. E. , Hippler, M. , Mitchell‐Olds, T. , Gershenzon, J. , & Vogel, H. (2004). Successful herbivore attack due to metabolic diversion of a plant chemical defense. Proceedings of the National Academy of Sciences of the United States of America, 101(14), 4859–4864. 15051878 10.1073/pnas.0308007101 PMC387339

[ece370086-bib-0054] Yin, B. , Li, G. , Wan, X. , Shang, G. , Wei, W. , & Zhang, Z. (2017). Large manipulative experiments reveal complex effects of food supplementation on population dynamics of Brandt's voles. Science China. Life Sciences, 60(8), 911–920. 28755298 10.1007/s11427-017-9114-9

[ece370086-bib-0055] Yu, R. P. , Zhang, W. P. , Yu, Y. C. , Yu, S. B. , Lambers, H. , & Li, L. (2020). Linking shifts in species composition induced by grazing with root traits for phosphorus acquisition in a typical steppe in Inner Mongolia. Science of the Total Environment, 712, 136495. 31945536 10.1016/j.scitotenv.2020.136495

[ece370086-bib-0056] Zhang, L. , Zhu, Z. , Li, Y. , Qian, Z. , Liu, G. , & Wang, X. (2019). Overall grazing tolerance index (overall GTI) is not an ideal predictor for describing a single‐species tolerance to grazing. Ecology and Evolution, 9(7), 4087–4102. 31015990 10.1002/ece3.5038 PMC6468136

[ece370086-bib-0057] Zhang, R. , Schellenberg, M. P. , Han, G. , Wang, H. , & Li, J. (2018). Drought weakens the positive effects of defoliation on native rhizomatous grasses but enhances the drought‐tolerance traits of native caespitose grasses. Ecology and Evolution, 8(23), 12126–12139. 30598805 10.1002/ece3.4671 PMC6303709

[ece370086-bib-0058] Zhang, X. , van Doan, C. , Arce, C. C. M. , Hu, L. , Gruenig, S. , Parisod, C. , Hibbard, B. E. , Hervé, M. R. , Nielson, C. , Robert, C. A. M. , Machado, R. A. R. , & Erb, M. (2019). Plant defense resistance in natural enemies of a specialist insect herbivore. Proceedings of the National Academy of Sciences of the United States of America, 116(46), 23174–23181. 31659056 10.1073/pnas.1912599116 PMC6859362

[ece370086-bib-0059] Zvereva, E. L. , Paolucci, L. N. , & Kozlov, M. V. (2020). Top‐down factors contribute to differences in insect herbivory between saplings and mature trees in boreal and tropical forests. Oecologia, 193(1), 167–176. 32314043 10.1007/s00442-020-04659-z PMC7235072

